# Supplementary material for: Investigating the influence of physiologically relevant hydrostatic pressure on CHO cell batch culture
Source: Sci Rep. 2021 Jan 8;11:162. doi: 10.1038/s41598-020-80576-8 (PMC7794228; doi:10.1038/s41598-020-80576-8)
Supplement: Supplementary file 1 — Supplementary Information 1. [file 41598_2020_80576_MOESM1_ESM.zip › S1/IgG/Feb17 60 90 standard/Feb15 90mmHg.pdf]

===

Acq. Operator : SYSTEM Seq. Line : 34
Sample Operator : SYSTEM
Acq. Instrument : HPLC-0039 Location : 6
Injection Date : 2/17/2019 4:26:24 PM Inj : 1
Inj Volume : 100.000 µl
Method : C:\Users\Public\Documents\ChemStation\1\Data\TK_CHO_IgG 2019-02-17 14-04-01\CHO_IgG_LC.M (Sequence Method)
Last changed : 2/8/2019 3:17:23 PM by SYSTEM

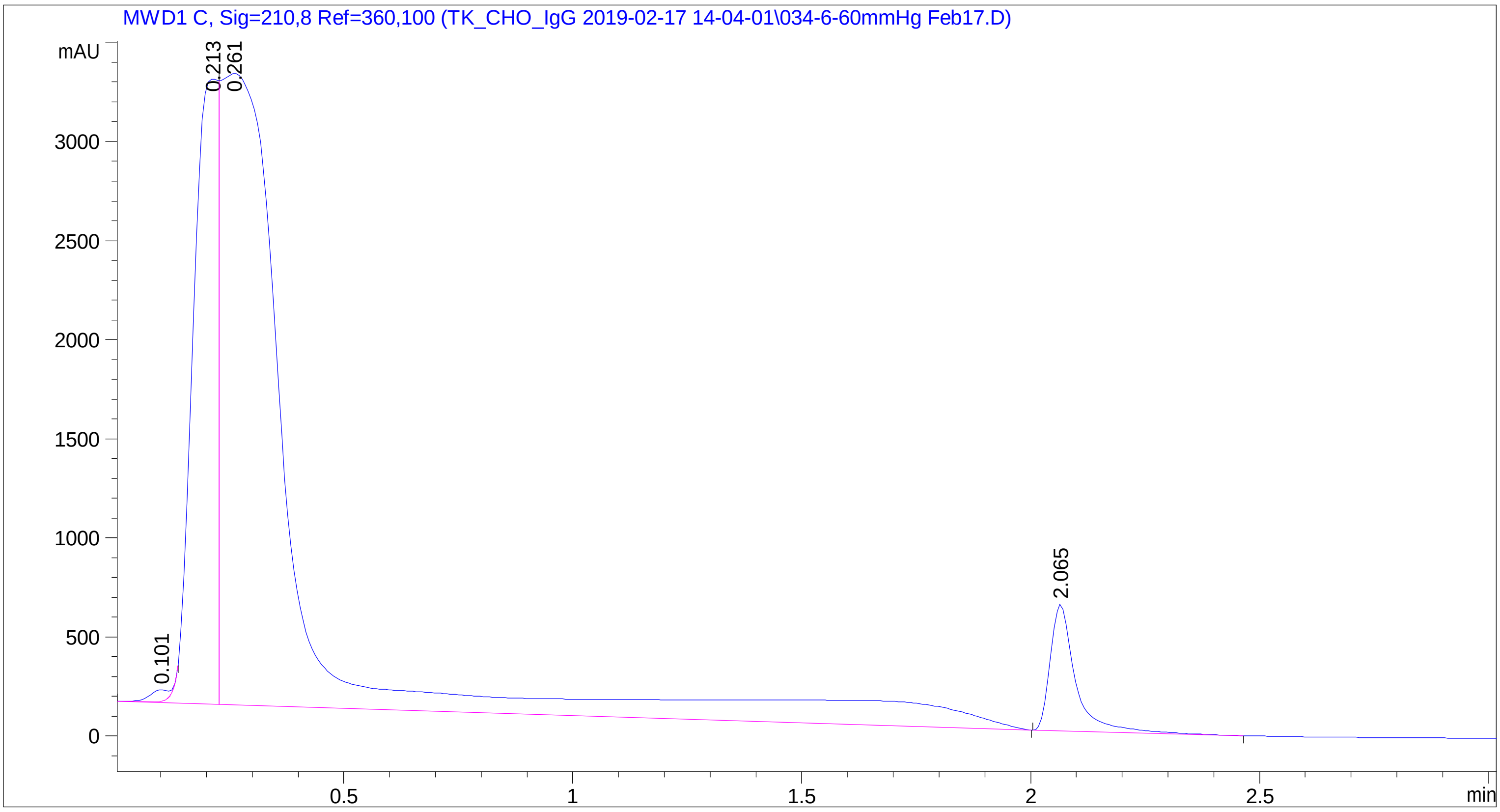

===

Area Percent Report

===

Sorted By : Signal
Multiplier : 1.0000
Dilution : 1.0000
Use Multiplier & Dilution Factor with ISTDs

Signal 1: MWD1 C, Sig=210,8 Ref=360,100

Peak #	RetTime [min]	Type	Width [min]	Area [mAU*s]	Height [mAU]	Area %
1	0.101	BV E	0.0406	165.49663	59.23054	0.3366
2	0.213	VV R	0.0603	1.17245e4	3154.23901	23.8428
3	0.261	VB	0.1596	3.48340e4	3184.11914	70.8380
4	2.065	BBA	0.0568	2450.22485	638.35193	4.9827

Totals : 4.91743e4 7035.94062

===

*** End of Report ***
